# Water Quality Sensing and Spatio-Temporal Monitoring Structure with Autocorrelation Kernel Methods

**DOI:** 10.3390/s17102357

**Published:** 2017-10-16

**Authors:** Iván P. Vizcaíno, Enrique V. Carrera, Sergio Muñoz-Romero, Luis H. Cumbal, José Luis Rojo-Álvarez

**Affiliations:** 1 Departamento de Eléctrica y Electrónica, Universidad de las Fuerzas Armadas ESPE, Av. General Rumiñahui s/n, Sangolquí 171-5-231B, Ecuador; evcarrera@espe.edu.ec; 2 Departamento de Teoría de la Señal y Comunicaciones y Sistemas Telemáticos y de Computación, Universidad Rey Juan Carlos, Camino del Molino s/n, 28943 Fuenlabrada, Spain; sergio.munoz@urjc.es (S.M.-R.); joseluis.rojo@urjc.es (J.L.R.-Á.); 3 Center for Computational Simulation, Universidad Politécnica de Madrid, 28223 Pozuelo de Alarcón, Spain;; 4 Centro de Nanociencia y Nanotecnología, Universidad de las Fuerzas Armadas ESPE, Av. General Rumiñahui s/n, Sangolquí 171-5-231B, Ecuador; lhcumbal@espe.edu.ec

**Keywords:** water quality, pollution measurements, spatio-temporal interpolation, support vector regression, Mahalanobis kernel, autocorrelation kernel

## Abstract

Pollution on water resources is usually analyzed with monitoring campaigns, which consist of programmed sampling, measurement, and recording of the most representative water quality parameters. These campaign measurements yields a non-uniform spatio-temporal sampled data structure to characterize complex dynamics phenomena. In this work, we propose an enhanced statistical interpolation method to provide water quality managers with statistically interpolated representations of spatial-temporal dynamics. Specifically, our proposal makes efficient use of the *a priori* available information of the quality parameter measurements through Support Vector Regression (SVR) based on Mercer’s kernels. The methods are benchmarked against previously proposed methods in three segments of the Machángara River and one segment of the San Pedro River in Ecuador, and their different dynamics are shown by statistically interpolated spatial-temporal maps. The best interpolation performance in terms of mean absolute error was the SVR with Mercer’s kernel given by either the Mahalanobis spatial-temporal covariance matrix or by the bivariate estimated autocorrelation function. In particular, the autocorrelation kernel provides with significant improvement of the estimation quality, consistently for all the six water quality variables, which points out the relevance of including *a priori* knowledge of the problem.

## 1. Introduction

Environmental pollution is related to the voluntary or involuntary inclusion of natural or artificial materials and substances that damage the ecosystem. Pollution of water resources is mainly due to the increment in population and industrial density [1]. Growing population waste poses a threat to public health and put in danger the continuous use of water reserves [2]. Typically, urban wastewater is a complex mixture containing water (usually over 99%) mixed with organic and inorganic compounds, both in suspension and dissolved with small concentrations [3]. In order to prevent public health problems and manage water resources, it is highly relevant to know the physical and chemical characteristics of such water resources.

Monitoring water quality of hydraulic resources through monitoring campaigns is one of the most important tasks in order to responsibly manage the use of water. Water quality monitoring focuses on programmed sampling, measurement, and recording of the most representative water quality parameters. Measurements are not usually taken uniformly at determined locations and times during the monitoring campaigns, and usually the pollutant concentrations in river waters do not follow linear variations [4]. It has been pointed out in previous works [5] that the representation of sensed data in different locations and different campaigns can provide useful information. In our previous work [6], we showed that it was possible to extract such information using conventional interpolation methods. However, those reconstruction methods based just on measures are extremely general and they do not consider the higher order statistical information of the available data in order to construct these representations.

In this work, we propose to use machine learning techniques based on advanced kernel methods to maximize the extracted information from the expensive and costly water quality measurement campaigns. The proposed methods allow to include statistical information of higher order in the models, especially the spatio-temporal covariance, through the Mahalanobis distance, and the spatio-temporal correlation, through the autocorrelation function of different consecutive sensors and over time. Both metrics are naturally included for their use as Mercer’s kernel for Support Vector methods, and they have been proposed and successfully used in other areas [7,8].

The rest of this paper is organized as follows. In Section 2, the materials and methods are explained, including a short mathematical description of the spatio-temporal interpolation algorithms and details of the database used for this analysis. In Section 3, results are presented for a number of measured variables, the algorithmic performance is benchmarked, and the analysis in several environmental variables and their spatio-temporal dynamics are visualized. The result discussion and the main conclusions are presented in Section 4.

## 2. Materials and Methods

### 2.1. Experimental Data

**Figure 1:**
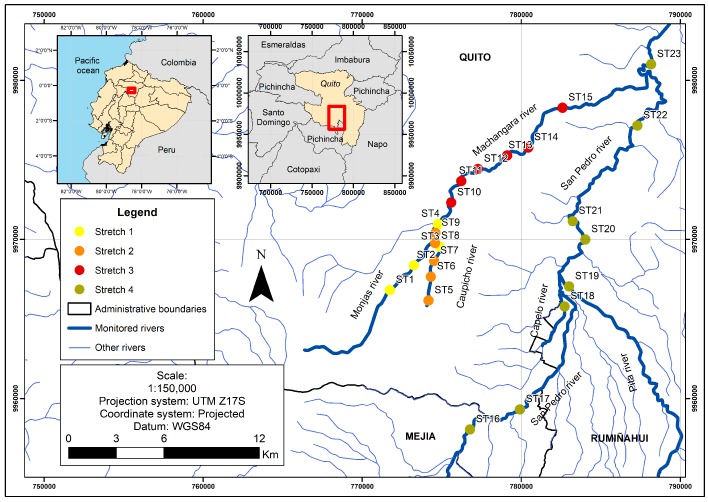
Location of monitoring stations at Machángara (Stretches 1, 2, and 3) and San Pedro (Stretch 4) Rivers. The station names and numeric codes were provided by the Metropolitan Water Company.

The dataset used in this work was provided by EPMAPS (Metropolitan Water Company of Quito, capital of Ecuador) and includes 15 water quality parameters of Machángara and San Pedro Rivers that were measured between 2002 and 2007 through 64 monitoring campaigns [9]. Located at about 2815 m above sea level, Machángara River is the main collector of wastewater of Quito, as far as it receives about 75% of the industrial and human waste [9,10]. Machángara River crosses the southern area of Quito with an approximate length of 22 km, where 25 water quality monitoring stations are located (see Figure 1). On the other hand, San Pedro River crosses the East of Quito through a population zone at about 2000 m above sea label, being the principal waste collector of that zone.

In this work, we have chosen three sections of the Machángara River which pass through three different population areas. The first and second areas are to the south of Quito and they are characterized by having a high population density and sharing their space with the industrial sector, whereas the third one is at the center of Quito, and it exclusively represents a commercial and touristic area. The fourth stretch corresponds to a section of the San Pedro River that crosses the East of Quito, which is mostly a housing and commercial area.

The monitoring stations used in this work are shown in Figure 1 and the description of the stations is presented in Table 1.

### 2.2. Sensors Description and Measurement Process

The procedures for water quality measurement used by the Metropolitan Water Company are intended for domestic, industrial and farming wastewater. Therefore, these procedures can be diverse when considering different sources of water pollution, and different water quality variables to be evaluated. Technicians of EPMAPS used measurement methods that can generally be applied to the three types of sources of pollution described above. The procedures used are given in the American Public Health Association Standard Methods [11], for temperature (2550 method), for dissolved oxygen (4500-O method), for chemical oxygen demand (5220-A method), and for biochemical oxygen demand (5210 method). The equipments used for measurements were: for flow rate (Q), a portable sampler ISCO 6712 which uses ultrasonic sensors; for DO, a portable YSI 58 Dissolved Oxygen Instrument with 5740 cable and 5739 probe; and for COD, a Hanna HI839800 COD Test Tube Heater. For water sampling, a YSI WS705 single-bottle composite/discrete water sampler was used. It was released into the river and recovered it every 24 h [12] and a 3.7 L plastic container for Biochemical Oxygen Demand and Chemical Oxygen Demand was taken. Water samples were preserved on ice and transported in a cooled box to the EPMAPS quality control laboratory.

The six water quality parameters selected as more relevant to be analyzed in this work are described and summarized in Table 2. The measurements of these parameters were obtained in campaigns separated by different time periods, and in not-equally-separated places along the river. Therefore, the measurements corresponded to a non-uniform spatio-temporal sampling grid that requires subsequent and careful digital processing.

### 2.3. Conventional Algorithms and Spatio-Temporal Interpolation

The EPMAPS database can entail some measurement errors caused by the inherent processes of capturing river water samples, transportation from field to laboratory, conservation of samples, response time for used chemical reagents, procedural and human errors, among others. These measurement errors of water quality parameters can be evidenced as non-intuitive spatio-temporal variations or as inherent noise of the measurement process. Even altered by noise, data convey valuable information that can be extracted with mathematical procedures of digital smoothing over the obtained spatio-temporal series. Two different kinds of interpolation algorithms had been previously proposed for this type of problems [6], namely, parametric algorithms (Delaunay linear and nearest), which do not require tuning parameters, and non-parametric algorithms (such as *k* Nearest Neighbors or *k-NN*), which require tuning some few free parameters. Algorithm *k-NN* had allowed to obtain the lowest absolute interpolation error (MAE) in comparison with other methods like Delaunay linear and nearest [6]. Therefore, we use *k-NN* again in this work as our reference algorithm.

The *k-NN* algorithm is a powerful statistical learning tool, widely and commonly used in data classification and regression. In addition, it is easy to implement in software, providing with robustness in the estimation, specially when used it in conjunction with cross-validation techniques [13]. The estimation process is accomplished by using only those few data closer to the target variable or test point $x_{e}$. For this purpose, a weighted function is used for each close neighbor $x_{i}$ and their respective distances to $x_{e}$. The most widely used distances in this setting are the Euclidean, the Manhattan, the Minkowski, the weighted Euclidean, the Mahalanobis, and the Cosine distances. The Mahalanobis distance between two points $x_{1}$ and $x_{2}$ is defined as

(1) $$dist_{M}\left(x_{1},x_{2}\right)=\sqrt{{\left(x_{1}-x_{2}\right)}^{T}\;\Sigma_{x}^{-1}\;\left(x_{1}-x_{2}\right)}$$

where Σx is the estimated covariance matrix of the available dataset. The Mahalanobis distance has advantageous properties when compared to the Euclidean distance, namely, it is invariant to changes in scale, it does not require previous normalization, and it does not depend on the measurements units. By using the matrix $\Sigma_{x}^{-1}$, we consider the covariance between variables and possibly the redundancy effect. The estimation function of $x_{e}$ is represented by $\hat{f}\left(x_{e}\right)$, and it is estimated according to the Distance Weighted Nearest Neighbor algorithm [14] as follows:

(2) $$\hat{f}\left(x_{e}\right)=\frac{\sum_{i=1}^{k}w_{i}f\left(x_{i}\right)}{\sum_{i=1}^{k}w_{i}}$$

where $f(x_{i})$ represents the value of *f* at that sample near to $x_{e}$, and $w_{i}$ are the weights that are defined in terms of the Mahalanobis distance as

(3) $$w_{i}=\frac{1}{dist_{M}{\left(x_{e},x_{i}\right)}^{2}}$$

Therefore, the interpolation algorithm is refined by weighing the contribution of each of the *k* neighbors according to their distance to point $x_{e}$, giving larger weights to the nearest neighbors. Whenever $x_{e}$ exactly matches one of $x_{i}$, the denominator becomes zero, and in that case we assign $\hat{f}\left(x_{e}\right)$ to be just $f(x_{i})$.

### 2.4. Support Vector Regression and Autocorrelation Kernel

One of the main contributions of the present work is the usage of kernel methods for interpolating, building, and visualizing the spatial-temporal dynamics of the measured variables. In this setting, kernel methods can be advantageous applied by taking into account the statistical structure of the variables [15,16].

Probably the most known Support Vector Machine (SVM) algorithm is the classification algorithm. However, increasing advantages have been obtained from the SVM algorithm for the nonlinear regression paradigm. The main differences between SVM classification and support vector regression (SVR) are the noise models and the loss functions. Vapnik proposed to use the ϵ-insensitive loss function for yielding sparse solutions in the SVR algorithm [8,17]. Let ϵ>0, we can define

(4) $$\ell \left(u\right)={\left|u\right|}_{\epsilon }=\left\{\begin{array}{cc} 0, & |u|<\epsilon \\ |u|-\epsilon , & \mathrm{otherwise} \end{array}\right.$$

This loss function assigns zero loss to any error smaller than ϵ, and it also provides some robustness against outliers. The regression function estimates the true function by constructing a tube around it, which defines a margin outside that function, treating the deviation as noise. Accordingly, the SVR model used here for spatial-temporal water quality maps uses the following nonlinear regression model:

(5) $$\hat{y}=f\left(x\right)=\langle w,\varphi \left(x\right)\rangle +b$$

where φ(x) denotes a nonlinear transformation to a usually higher dimensional feature space, and *b* is the bias term. Now we consider a dataset $D\;=\;\left\{x_{1},y_{1}\right\},\;\ldots ,\left\{x_{N},y_{N}\right\}$, with $\;x\in R^{d},\;y\in R$, where *d* is the dimension of the input data, and *N* is the number of observed samples or measurements. Then, the ν-SVR algorithm states that the function to be minimized [18] is

(6) $$\frac{1}{2}{∥w∥}^{2}+C\left(\nu \epsilon +\frac{1}{N}\sum_{i=1}^{N}\left(\xi_{i}+\xi_{i}^{*}\right)\right)$$

where the first term is a $L_{2}$ regularization and the second term is the ϵ-insensitive loss function. Note that again ϵ is the insensitiveness parameter, *C* is another previously established parameter that allows us to adjust the trade-off between the error tolerance and the softness of the regression, $\xi_{i}$ and $\xi_{i}^{*}$ are the slack variables representing the excess of error for each sample $\left(x_{i},y_{i}\right)$, and ν is an operative parameter used to control the parameter ϵ in terms of the maximum deviation from the real value allowed at each measurement. Taking into account the following constraints,

(7) $$\xi_{i},\xi_{i}^{*}\geq 0,\;\forall i=1,\ldots ,N$$

(8) $$y_{i}-\left(\langle w,\varphi \left(x\right)\rangle +b\right)\leq \xi_{i}+\epsilon$$

(9) $$\left(\langle w,\varphi \left(x\right)\rangle +b\right)-y_{i}\leq \xi_{i}^{*}+\epsilon$$

and using the Lagrangian function, the solution to the nonlinear SVR is

(10) $$w=\sum_{i=1}^{N}\eta_{i}\varphi \left(x_{i}\right)$$

where $\eta_{i},\;i=1,2,3,\ldots ,N$ are scalars, and samples $x_{i}$ for which $\eta_{i}\neq 0$ are the support vectors. Thus,

(11) $$\hat{y}=f\left(x\right)=\langle w,\varphi \left(x\right)\rangle +b=\sum_{i=1}^{N}\eta_{i}\langle \varphi \left(x_{i},\varphi \left(x\right)\right)\rangle +b$$

which is the same as:

(12) $$\hat{y}=f\left(x\right)=\sum_{i=1}^{N}\eta_{i}K\left(x_{i},x\right)+b$$

where K(·,·) denotes a Mercer’s kernel, and it stands for the dot product in a high-dimensional space, without needing to explicitly know either the nonlinear transformation or the space.

In this work, we use the ν-SVR to provide the estimation of the support vectors and their number ($n_{SV}$) to be maintained in the solution with respect to the total number of samples in the dataset. Then, the solution can be linearly expressed in terms of the kernel function and the available samples. Among the most usual Mercer’s kernels, we find the linear and the Gaussian ones. Here, we follow an approach of increasing statistical knowledge about the data structure to be incorporated to the algorithm in order to provide with improved performance, as follows.

First, we use a SVR with the conventional Gaussian radial basis function kernel (*RBF-SVR*). In this case, the kernel is a bivariate function given by

(13) $$K\left(x_{i},x_{j}\right)=\exp\left(-\frac{1}{2\sigma^{2}}{∥x_{i}-x_{j}∥}^{2}\right)$$

where σ allows to control the neighborhood of samples on which each sample influences and contributes to create the solution. These models can approximate the underlying function of a wide variety of data as long as σ is tuned, usually by cross-validation. Note that in this case, we have radial symmetry and we assume that changes in both dimensions of our data (time and space) follow similar dynamics. However, temporal and spatial variations will probably be different, both physically and in terms of their units. Whereas normalization of input variables can alleviate this problem to some extent, other advanced kernels can be used instead, which do not need normalization.

Second, we propose to use a non-radially symmetric kernel, by using for this purpose the covariance matrix of the data, given by $\Sigma_{x}$. Hence, a SVR with a Mahalanobis distance kernel (*Ma-SVR*) is built, and in this case, the kernel equation is given by

(14) $$K\left(x_{i},x_{j}\right)=\exp\left(-\frac{1}{2}{x_{i}}^{T}\;\Sigma_{x}^{-1}\;x_{j}\right)$$

where the exponent considers the covariance-weighted distance between samples $x_{i}$ and $x_{j}$. Note that different spatial and temporal scales do not distort these distances, and neither does the use of different measurement units.

Third, we use a SVR with an Autocorrelation kernel (*Au-SVR*), as follows,

(15) $$K\left(x_{i},x_{j}\right)=\hat{R}\left(x_{i}-x_{j}\right)$$

This is a recently proposed new type of admissible SVR kernel, which uses the advantages of estimating the autocorrelation among samples. The autocorrelation function is highly relevant in signal processing, and it is a basic feature of random processes. One of the characteristics of the autocorrelation function is its symmetry in terms of the elements of the kernel matrix elements, $K\left(x_{i},x_{j}\right)=R\left(-\left(x_{i}-x_{j}\right)\right)=R\left(x_{i}-x_{j}\right)$. Recall that the autocorrelation is a solid measurement of the dependence of successive samples on previous ones [19], and for stationary processes, it depends only on the relative argument difference, and not on the absolute value of the argument.

We look for the optimum relationship between the amount of data, the approximation quality of the data by the chosen function of a set of functions, and the parameters that characterize that function set [20]. We use the SRM (Structural Risk Minimization) induction principle, which controls the approximation capabilities of a set of hypothesis functions in many different ways. The simplest interpretation of this approach is that: (1) it has the smallest number of features (free parameters); (2) it has the smallest algorithmic complexity; and (3) it has the largest margin. The problem is to find the best SVR structure that allows us to obtain algorithms which can generalize the data and measurements structure in the presence of noise for our data.

**Figure 2:**
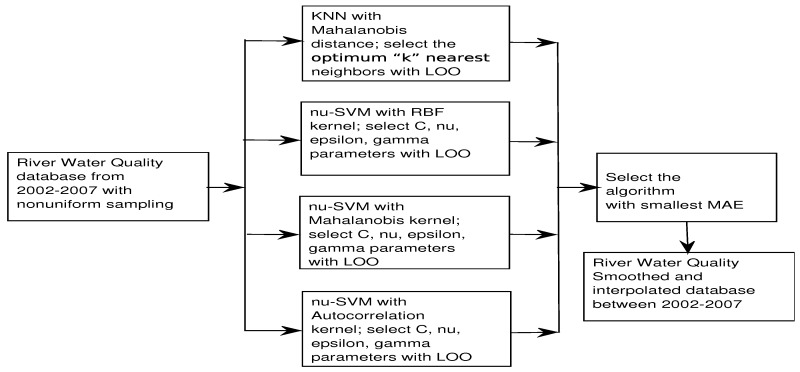
Flowchart of smoothing/interpolation for river water quality parameters.

The interpolation methods were evaluated using cross-validation trough LOO (Leave One Out) to obtain the MAE. Figure 2 shows a summary scheme of smoothing and interpolation processes used in this work.

## 3. Results

The next subsection summarizes results mostly on the performance of each benchmarked algorithm, which allows us to identify the best algorithms to be subsequently used. After this, a detailed analysis is made on the dynamics of the selected water quality variables according to the two best algorithms, in order to trust not only a single algorithm when drawing conclusions on the dynamics, and to check that we are analyzing actual spatio-temporal dynamics, rather than possibly-occurring interpolation artifacts due to the algorithm at hand. We used ${\mathrm{Matlab}}^{\mathrm{TM}}$ to implement our own software for most of the methods, which provides us with better control on the free parameter tuning and many other technical details.

### 3.1. Spatio-Temporal Interpolation Performance

According to Table 1, Stretch 1 contains the lowest number of observations during the 2002 to 2007 sampling period. This is due to its smaller number of monitoring stations when compared to the other analyzed areas; in addition, the length between the first and last stations is the smallest one. However, the number of stations does not necessarily increase with the travel distance of the stretch, as it can seen between Stretches 3 and 4, because of the different priority of water quality monitoring given by EPMAPS to different spatial locations. For instance, Stretch 3 contains the largest number of monitoring stations, although its travel distance is not the longest one, because this river section collects the last human and industrial waste from Quito before these waters take a different course from the city and flow to less populated areas.

**Figure 3:**
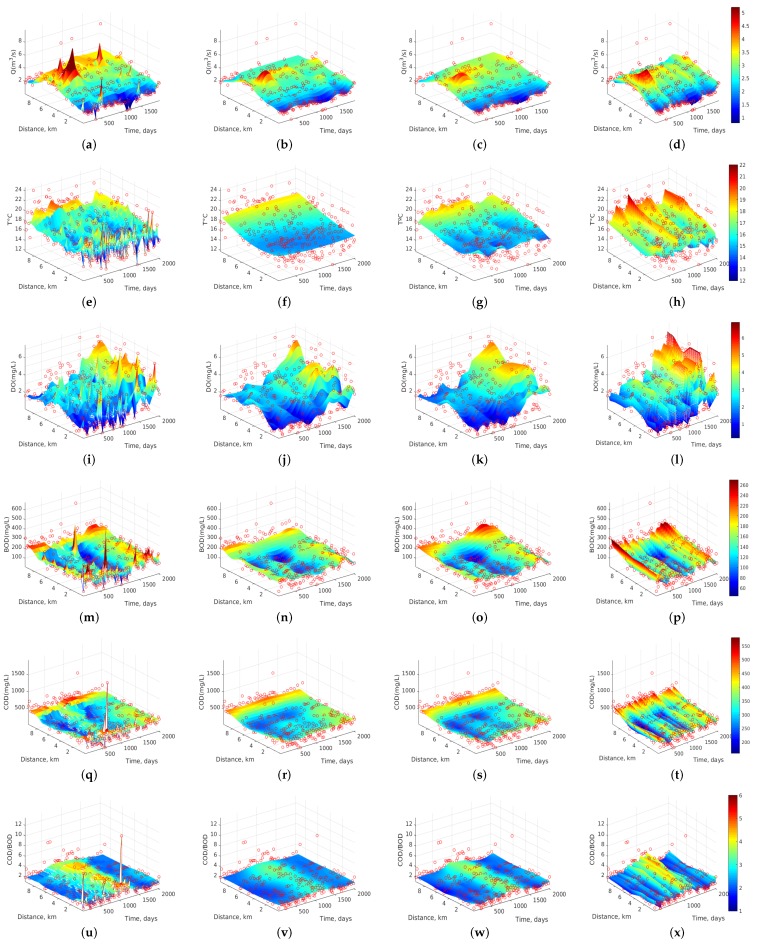
Dynamics of water quality variables for Stretch 3. From left to right (columns), results with *k-NN*, *RBF-SVR*, *Ma-SVR*, and *Au-SVR* algorithms, for the selected variables: (**a**–**d**) Q in m${}^{3}$/s; (**e**–**h**) T in ${}^{\circ }$C; (**i**–**l**) DO in mg/L; (**m**–**p**) BOD in mg/L; (**q**–**t**) COD in mg/L; and (**u**–**x**) COD/BOD ratio (dimensionless).

Figure 3 shows the dynamics of 6 water quality variables of Stretch 3 whose analysis will be discussed in more detail in Section 3.2. Table 3 shows the MAE interpolation errors obtained when *k-NN*, *RBF-SVR*, *Ma-SVR*, and *Au-SVR* algorithms are used on the dataset. The free parameters of these algorithms were adjusted by using a leave-one-out cross-validation procedure. That table shows that *k-NN* presents the largest MAE with an average of 32.1, followed by 28.0 for *RBF-SVR* and *Ma-SVR*. The lowest value is obtained by the *Au-SVR* algorithm, with an average MAE of 25.6. Note that *RBF-SVR* and *Ma-SVR* often exhibit similar MAE values in most of the variables, though the computational cost for optimizing the free parameters of the *RBF-SVR* algorithm is much lower than in the *Ma-SVR* algorithm. The lowest value of MAE in Q corresponds to the Stretch 1, this is possibly due to the fact that at the sites of flow measurement there has been no transient storage of water and the measurements lack considerable temporal variations of water flow. MAEs in T are similar (about 1.2) for all the analyzed stretches, and a similar behavior can also be observed with the MAE of the DO (about 0.7).

In contrast, there are large differences in the MAE of BOD, which is minimum (7.63) for Stretch 4 and it reaches its maximum (105.36) for Stretch 1. Similar behavior can also be observed in the MAE of COD, since it is minimum (24.3) for Stretch 4 and maximum (238.65) for Stretch 1. Since BOD and COD are related through the biodegradability index (COD/BOD), its MAE is approximately constant (0.55) for the the Machángara River stretches, but that is not true for the San Pedro River stretch, whose value is practically doubled. It is reasonable to find this difference since they are two different rivers.

Whereas the performance of the spatio-temporal interpolation methods can be benchmarked in terms of MAE, this represents a global performance measurements. However, it is highly desirable to have more detailed performance measurements for each interpolated segment, in order to complement the global ones. We further scrutinized the interpolated models in terms of Bland-Altman residual plots [21]. In this representation, the residuals of a given model are represented as a function of the measured magnitude, which provides us with a statistical representation of the residual distribution and allows to identify variance distribution and presence of model bias. For these plots being representative of the actual model quality, residuals were obtained on an out-of-sample approach, this is, the model is built for every sample except one, and then, the prediction of the excluded sample is obtained, and its residual is calculated. Repeating this process for all the samples gives a solid estimation of the model residuals.

On the other hand, given that the interpolation algorithms work on a spatio-temporal domain, the distribution of the residuals in this domain is also relevant when benchmarking different methods, as proposed in previous works [6]. Taking into account that the leave-one-out residual is obtained for each method in each sample, spatio-temporal residual plots show the difference in terms of Absolute Error (AE) between method *A* and method *B*, for two example variables. Blue markers represent the difference of AE ($\Delta AE=AE_{A}-AE_{B}$) when method *A* obtains worst performances than method *B* (i.e., for the case $AE_{A}-AE_{B}>0$), and red markers are shown otherwise (i.e., for the case $\Delta AE=AE_{A}-AE_{B}$< 0).

**Figure 4:**
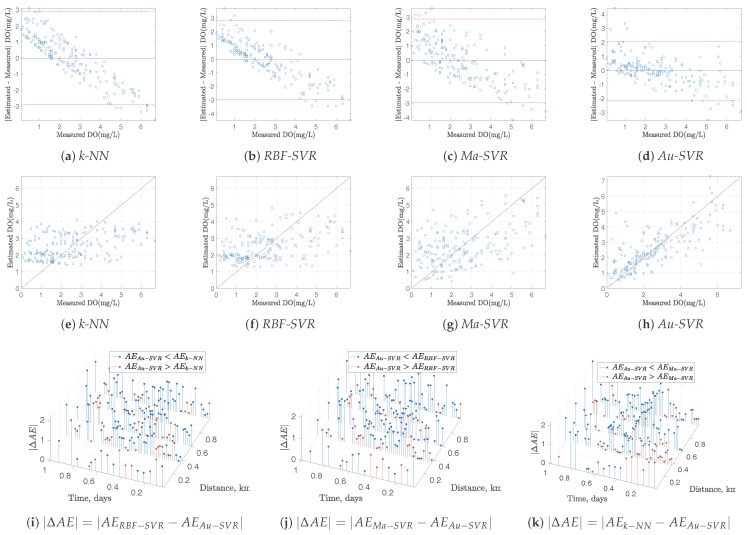
Residual analysis for DO in Stretch 2: (**a**–**d**) Bland-Altman plots; (**e**–**h**) Scatter plots; (**i**–**k**) |ΔAE| of Au−SVM compared with the other methods.

**Figure 5:**
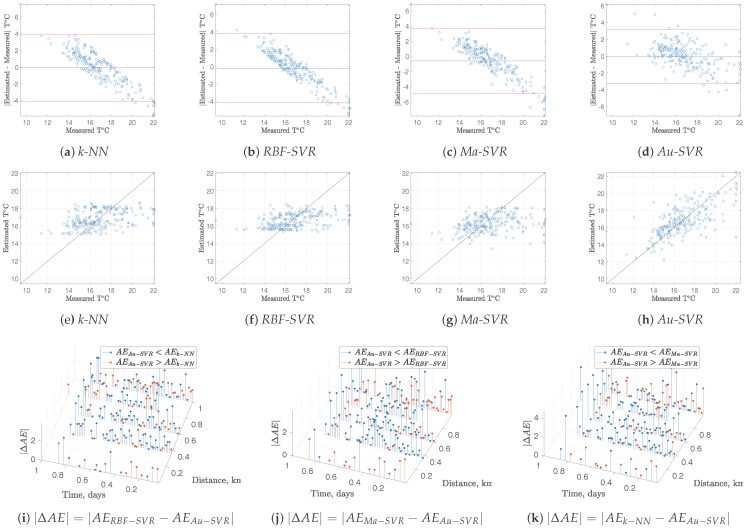
Residual analysis for T in Stretch 2: (**a**–**d**) Bland-Altman plots; (**e**–**h**) Scatter plots; (**i**–**k**) |ΔAE| of Au−SVM compared with the other methods.

Figure 4 and Figure 5 depict all these diagnostic plots for DO and T measurements, respectively, together with the scatter plots of the measured and estimated variables with the different methods. Bland-Altman plots show a strong model bias with *k*-NN and RBF-SVR, and this is seen in the scatterplots as stiffness in the model to follow the variations in the measurements. However, this trend is smoothed both for Ma-SVR and for Au-SVR, being the last one the best in terms of lower model bias and improved scatter. Spatio-temporal plots of ΔAE also reveal the predominance of increased performance of Au-SVM compared with the other methods, which can be seen on the predominant presence of blue markers for the absolute residuals in all cases. This explanation is valid both for DO and T data models.

### 3.2. Dynamics Analysis of Water Quality Measurements

**Figure 6:**
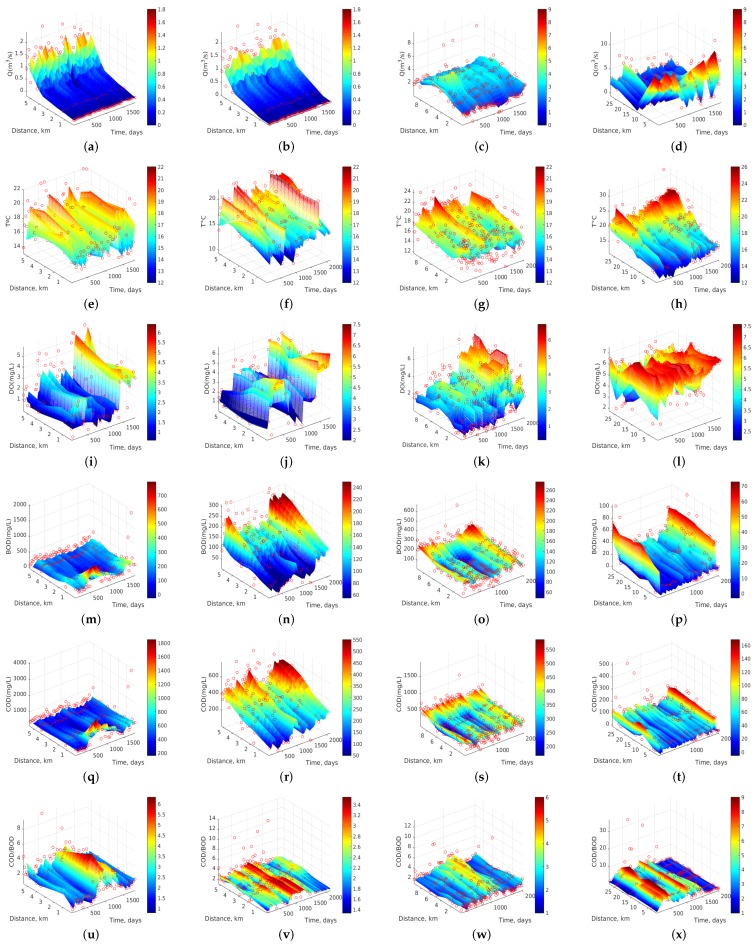
Dynamics of water quality variables for Stretches 1, 2, 3, and 4 (from left to right), when they are smoothed with the *Au-SVR* algorithm in terms of the estimated autocorrelation of the available data: (**a**–**d**) Q in m${}^{3}$/s; (**e**–**h**) T in ${}^{\circ }$C; (**i**–**l**) DO in mg/L; (**m**–**p**) BOD in mg/L; (**q**–**t**) COD in mg/L; and (**u**–**x**) COD/BOD ratio (dimensionless).

**Figure 7:**
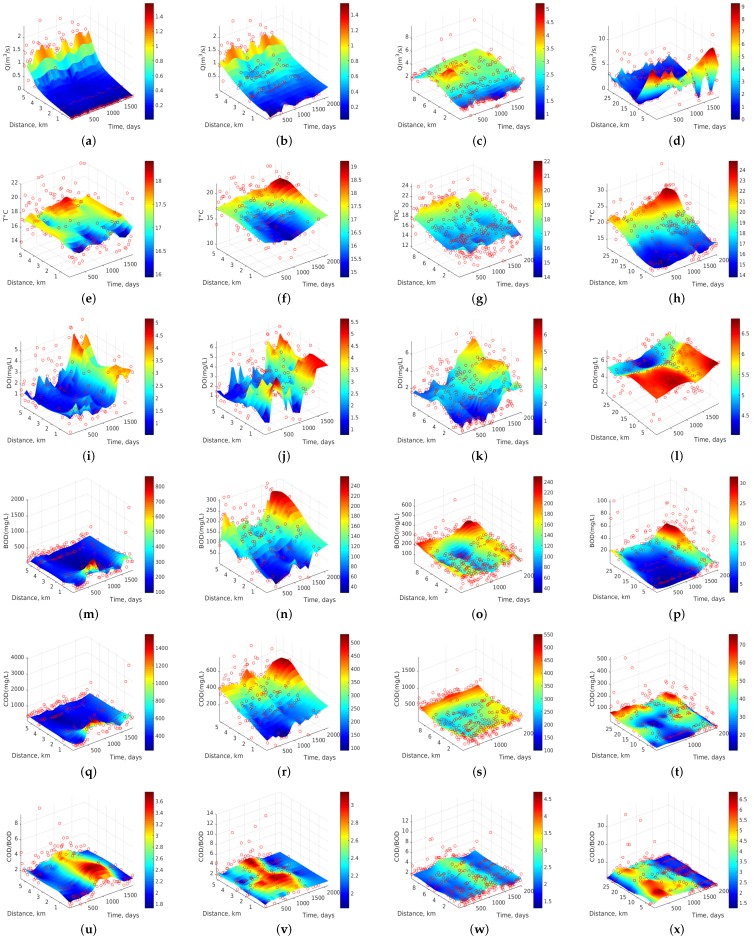
Dynamics of water quality variables for Stretches 1, 2, 3, and 4 (from left to right), when they are smoothed with the *Ma-SVR* algorithm in terms of the Mahalanobis distance from data covariance: (**a**–**d**) Q in m${}^{3}$/s; (**e**–**h**) T in ${}^{\circ }$C; (**i**–**l**) DO in mg/L; (**m**–**p**) BOD in mg/L; (**q**–**t**) COD in mg/L; and (**u**–**x**) COD/BOD ratio (dimensionless).

In this section, we first scrutinize in detail the dynamics of Stretch 3 according to all the algorithms (Figure 3), and then we study all the stretches with the two best algorithms according to the previous section, namely *Au-SVR* (Figure 6) and *Ma-SVR* (Figure 7).

Figure 3 shows the spatio-temporal dynamics of the six water quality variables of Stretch 3 for all the algorithms. We have chosen the representation of this stretch because it belongs to the final section of the most important river (i.e., Machángara) in Quito. Then, after monitoring station ST15, the river leaves the city and moves to less populated areas. In Figure 3a–d, Q dynamics is represented as estimated by each interpolation algorithm, showing in all of them a trend to increase the flow rate, with a maximum value of about 5 m${}^{3}$/s at about 7 km (ST14) and at day 600 (July 2002). This trend is maintained for the entire sampling period (2002–2007) and it can be more clearly seen in the last 3 algorithms. Figure 3e–h shows the dynamics of T at Stretch 3, revealing that changes in T are not smooth with the *k-NN* algorithm compared to *Ma-SVR* and *Au-SVR* algorithms, indicating that this algorithm intends to follow the atypical measurements without fully catching the dynamics. On the other hand, *RBF-SVR* softens too much the estimated dynamics, while *Ma-SVR* and *Au-SVR* show similar and better smoothness of spatio-temporal trends. As observed, T trends to rise at the last monitoring stations, possibly because they collect more human and industrial wastewater than the other ones. Also, every about 500 days (15 months) T rises up to its maximum value (between 19 ${}^{\circ }$C and 21 ${}^{\circ }$C), which could indicate periodicity or near-seasonal change affecting to the Quito temperature. Figure 3i–l shows the dynamics of DO. A similar trend was observed for all the algorithms in the DO concentration between days 1500 and 2025 (August 2004 to January 2007), where DO reaches near 6.5 mg/L and there is an increase of DO up to this maximum value from 3 km and downstream. This can be interpreted taking into account that the last 6.49 km of this section (total distance of 9.49 km) include topographical changes in the river course, which cause strong tapping of water over the rocks, and this effect could help to increase the DO concentration.

Figure 3m–p shows the interpolation results for BOD, revealing very well-defined spatial and temporal trends for the three SVR algorithms, whereas *k-NN* turns to be extremely sensitive to atypical observations. During the time span of 800 to 1300 days (December 2003 to February 2005), the BOD concentration decreased to about 70 mg/L, and there was an increment of BOD in the last two monitoring stations (at 7 and 9 km). Figure 3q–t shows the COD estimated dynamics, with similar accuracy shown by the algorithms to the BOD case. Also, COD concentration at 9 km (ST15) is kept at approximately 540 mg/L for the entire sampling period. Figure 3u–x shows the dynamics of the biodegradability index (COD/BOD). It is clearly observed that, from 900 to 1300 days (March 2004 and February 2005), this parameter increased to 4.3 and it remains almost constant on the monitoring stations from ST10 to ST15. Recall that this index can describe the organic matter biodegradability according to the following ranges [22,23]:

- If COD/BOD ≤ 2.5, then the organic matter is very degradable.
- If COD/BOD ∈ (2.5, 5), then the organic matter is moderately degradable.
- If COD/BOD ≥ 5, then the organic matter is little degradable.

Therefore, this river section can be classified as moderately degradable, while for the other time spans beyond this range, the COD/BOD index is averaged to 2.1, meaning that organic matter is then highly degradable.

Now, we study the dynamics of all the variables in all the stretches, by looking at the two best algorithms so far, namely *Au-SVR* and *Ma-SVR*. In Figure 6, panels (a,b) exhibit an increase in Q at the last monitoring stations of each section during the 5-year observation, pointing at the gradual increase of human and industrial waste. In contrast, panel (c) shows an increasing Q to about 5 m${}^{3}$/s between 4 and 7 km (ST13 and ST14). Panel (d) shows a different behavior, as Q is maximum (up to 8 m${}^{3}$/s) for the first 8 km, and then it decreases near to 1.5 m${}^{3}$/s, which may be associated to the use of some water to irrigate crops in this spatial section of San Pedro River. Figure 6e–h shows the T dynamics in the four studied sections. In general, a trend to increased T is observed at the last station of each river section for the 5 years of monitoring. In panels (a,b,c), some periodicity of increasing T can be detected for all stations and for each year. Figure 6i–l shows the variations in DO. Panels (a,b,c) exhibit an increase in DO during the last two years (2006–2007), while panel (d) presents a different behavior with greater DO at the beginning of each river section and year, due to the abrupt topography.

Figure 6u–x shows the variations of the biodegradability index (COD/BOD) in the four river sections. The first panel shows an index increment up to 6 for the time period between 800 and 1000 days (December 2003 to June 2004), and from 0.5 to 2.5 km. The second panel shows an index increment up to 3.4 between 820 and 1400 days (January 2004 to May 2006) for all the monitoring stations. The time period in which this increment occurred was after the increase of the previous period and section. The third panel shows the index dynamics of Stretch 3, where an increment is observed between 820 and 1400 days (January 2004 to May 2006) for all monitoring stations. This time period in which the COD/BOD rises is similar to the time period of Stretch 2, which could be expected since Section 3 is its continuation in the same river. The fourth panel shows a different behavior regarding to the temporal trends in the different sections. From 200 to 600 days (June 2002 to May 2003), the biodegradability index rise to an alarming level, up to 9, showing a non-degradable water-type. These increments reappear up to an 8 value, from days 1000 to 1100 (June 2004 to September 2004), however, the index decreases to 1 (i.e., very degradable) after 1580 days (November 2005). Then, it remains near constant until the end of the sampling period in 2007.

We can contrast the previous results with the ones obtained with the *Ma-SVR* algorithm. For instance, in Figure 7a–d, the Q dynamics of the four stretches shows the same spatio-temporal trends as those in Figure 6. The behavior of T in Figure 7e–h seems more smoothed when compared to Figure 6, and some peaks turn to be smoother, as seen in Stretch 1 results. The variations of DO, BOD, COD, and COD/BOD, exhibit a similar smoothing behavior when using this kernel.

## 4. Discussion and Conclusions

A set of spatio-temporal interpolation methods have been benchmarked in order to determine their performance to estimate the measurement dynamics in water quality control campaigns. Emphasis has been made in including *a priori* information into the interpolation schemes.

**Algorithmic considerations.** From an algorithmic performance point of view, we can conclude that the changes in MAE are consistent for each stretch and for each variable when the same algorithm is used. The quality provided by *k-NN* and *SVR-RBF* is similar, just slightly better for the second one in some few variables. However, there is a consistent improvement when using *Ma-SVR* and *Au-SVR* algorithms when compared to the two previous ones, and there is also a consistent improvement when using *Au-SVR* even in comparison with *Ma-SVR*. These results imply that the use of kernel methods improves the existing interpolation techniques in these environments, and more, that the use of increasingly advanced statistical description of the spatio-temporal properties dramatically improves the dynamics estimation for all the variables. The use as Mercer’s kernels of the estimated autocorrelation of the data turns to be much more effective than the use of the Mahalanobis distance from the estimated covariance.

**Estimation and visualization of spatio-temporal dynamics.** The dynamics of water quality variables can be often hard to predict due to its very changing nature [24,25]. The spatio-temporal interpolation of measurements in different stations and in different campaigns has been previously shown not to be limited to a mere visualization, but instead it represents the estimation of the variable dynamics, which allows the researcher to get deep further knowledge into the relevant information conveyed in the already available data, such as temporal space trends or physical and chemical characteristics of pollutants that are closely related to environmental conditions and the geographical areas though which the stretches of the river circulate. In this work, 5-year databases were used, going further than previous works in the literature [26,27] which are constrained to data from 2 years. This represents an advantage in terms of the amount of data used for the study of spatio-temporal dynamics of the water quality variables. Therefore, graphical representations showed more reliable and consistent spatial and temporal trends in the dynamics. The quality of our database is affected by technological limitations, as previously pointed in the literature for this kind of data [25], thus, water quality databases contains both the dynamics and its own different types of noise of the measurement process.

**Kernel methods advantages.** The use of machine learning algorithms has been previously shown to be a key point in retrieving the dynamics from the measurements in campaigns [6], where classic interpolation algorithms were shown to extract the spatio-temporal dynamics from the available observations. The best classic interpolation algorithm was used in this work, namely *k-NN*, but it was shown to exhibit some estimation limitations. Whereas *k-NN* has been proven to be an excellent multidimensional interpolation algorithm in a number of applications [28,29,30], it is limited if we want to include *a priori* information available from our problem. This has motivated the proposal of kernel methods, and particularly, the proposal of SVR. The extremely good generalization capabilities of SVR brings improvement when using a general Mercer’s kernel like the RBF one [31,32]. But this work has gone further, and two ways of including the higher order statistical description of the observations have been scrutinized, namely, the estimated covariance matrix and the estimated autocorrelation matrix. The average MAE obtained in previous work [6] with respect to these six variables for Stretch 3 when using the *k-NN* algorithm was 28.1, whereas the use of SVR with estimated autocorrelation kernel yielded 20.5. The inclusion of this statistical data structure has proven to be significantly advantageous, any of them definitely improves the estimation quality, and the autocorrelation kernel yields always the best model in our data.

**Limitations of the study.** Our approach to the analysis of spatio-temporal dynamics in water quality measurements was necessarily constrained to the availability of measurements in conventional campaigns. Other richer data sources, as satellite monitoring images, could improve the data quality, but this is not possible for a good number of the measured variables. Sampling density clearly resulted in limited expression of spatio-temporal models, hence, increased sampling density would be desirable, both in time and space, and some technological possibilities for this purpose could include the use of wireless sensor networks. These data sources have not been covered in the present approach, though our findings could be readily applied when they become technologically more available.

The analysis of the water quality of three sections of the Machángara River and a section of the San Pedro River by means of non-parametric interpolation methods in a non-uniform sampling scenario has allowed us to obtain relevant information about the spatio-temporal dynamics of these stretches. The best interpolation method evaluated in terms of the MAE was the SVR with the estimated autocorrelation kernel, which points out the relevance of including *a priori* knowledge of the problem. The interpolation results partially solve the practical problem of the absence of water quality measurements in difficult-to-access places and for municipalities with a tight budget for carrying out more sampling campaigns. In addition, these results can provide with complementary information to public enterprises managing water for cities and fields, and better exploitation of the currently available measurements from campaigns.

## Figures and Tables

**Table 1:** Monitoring stations used in this work. Stretch 1 is Shanshayacu Ravine, Stretch 2 is Machángara River, Stretch 3 is El Trébol, and Stretch 4 is San Pedro River. Parameter *d* corresponds to the distance from each other station with respect to the first one in every studied stretch.

Studied Stretch	Station Number	Station Name	Code	*d* (km)
Stretch 1	ST1	Q. Shanshayacu	1.02	0.00
	ST2	Q. Ortega	1.04	1.30
	ST3	R. Mch. Quimiag	1.07	4.27
	ST4	R. Mch. Quito Sur	2.05	5.54
Stretch 2	ST5	R. Mch. Caupichu	2.01	0.00
	ST6	R. Mch. Oleoducto	2.02	1.89
	ST7	R. Mch. La Lucha	2.03	3.15
	ST8	R. Mch. Fosforera	2.04	4.27
	ST9	R. Mch. Quito Sur	2.05	5.25
Stretch 3	ST10	R. Mch. El Recreo	2.07	0.00
	ST11	R. Mch. Villaflora	2.08	1.75
	ST12	R. Mch. El Sena	2.09	2.75
	ST13	R. Mch. El Trébol	2.10	4.91
	ST14	R. Mch. Las Orquídeas	2.11	6.31
	ST15	Q. El Batán	1.09	9.49
Stretch 4	ST16	R. SP. Trópico	4.02	0.00
	ST17	R. SP Amaguaña	4.03	2.98
	ST18	R. SP Capelo	4.04	11.12
	ST19	R. SP Triángulo	4.06	12.55
	ST20	R. SP Guangopolo	4.07	16.09
	ST21	R. SP Guangopolo canal	4.09	17.23
	ST22	R. SP Cumbaya Cerv.	4.10	24.34
	ST23	R. SP AJ Machángara	4.13	28.42

**Table 2:** Studied water quality parameters for Stretches 1, 2, 3, and 4, in the case studies of Machángara and San Pedro Rivers.

Parameter	Acronym	Units
Flow rate	Q	m${}^{3}$/s
Temperature	T	${}^{\circ }$C
Dissolved Oxygen	DO	mg/L
Chemical Oxygen Demand	COD	mg/L
Biochemical Oxygen Demand	BOD	mg/L
COD/BOD ratio	COD/BOD	–

**Table 3:** Number of measurements (N. Meas.) and interpolation MAE for each variable by using *k-NN*, *RBF-SVR*, *Ma-SVR*, and *Au-SVR* methods for River Stretches 1, 2, 3, and 4. Bold numbers indicate the best performance among the methods.

Stretch	Variable	N. Meas.	k−NN	RBF−SVR	Ma−SVR	Au−SVR
Stretch 1	Q	177	0.11	0.11	0.10	**0.09**
	T	177	1.52	1.29	1.31	**1.27**
	DO	177	1.02	0.98	0.98	**0.81**
	BOD	177	124.54	105.99	108.65	**105.3**
	COD	177	283.75	244.81	246.28	**238.6**
	COD/BOD	177	0.68	0.64	0.65	**0.54**
Stretch 2	Q	212	0.15	0.15	0.14	**0.13**
	T	212	1.58	1.54	1.59	**1.20**
	DO	212	1.18	1.17	1.15	**0.75**
	BOD	212	38.41	37.96	37.52	**30.38**
	COD	212	92.69	88.86	86.77	**77.44**
	COD/BOD	212	0.90	0.80	0.82	**0.73**
Stretch 3	Q	306	0.58	0.48	0.49	**0.48**
	T	393	1.88	1.50	1.51	**1.38**
	DO	329	1.03	1.01	0.99	**0.74**
	BOD	396	49.14	40.96	40.15	**36.19**
	COD	396	114.96	94.82	94.24	**83.56**
	COD/BOD	396	0.79	0.65	0.65	**0.55**
Stretch 4	Q	303	1.07	1.05	0.96	**0.90**
	T	303	1.90	1.76	1.76	**1.30**
	DO	303	0.93	0.85	0.85	**0.71**
	BOD	303	12.02	10.89	10.89	**7.63**
	COD	303	38.34	32.48	32.23	**24.30**
	COD/BOD	303	2.01	1.74	1.74	**1.12**
	Average		32.13	28.02	28.02	**25.67**
